# Associations Among Genotype, Clinical Phenotype, and Intracellular Localization of Trafficking Proteins in ARC Syndrome

**DOI:** 10.1002/humu.22155

**Published:** 2012-07-02

**Authors:** Holly Smith, Romain Galmes, Ekaterina Gogolina, Anna Straatman-Iwanowska, Kim Reay, Blerida Banushi, Christopher K Bruce, Andrew R Cullinane, Rene Romero, Richard Chang, Oanez Ackermann, Clarisse Baumann, Hakan Cangul, Fatma Cakmak Celik, Canan Aygun, Richard Coward, Carlo Dionisi-Vici, Barbara Sibbles, Carol Inward, Chong Ae Kim, Judith Klumperman, A S Knisely, Steven P Watson, Paul Gissen

**Affiliations:** 1 Medical and Molecular Genetics, School of Clinical and Experimental Medicine, College of Medical and Dental Sciences, University of BirminghamBirmingham, United Kingdom; 2 Medical Research Council Laboratory for Molecular Cell Biology, University College LondonLondon, United Kingdom; 3 University College London Institute of Child Health, University College LondonLondon, United Kingdom; 4 Department of Cell Biology, University Medical CenterUtrecht, the Netherlands; 5 Medical School, Edinburgh UniversityEdinburgh, United Kingdom; 6 West Midlands Regional Genetics Laboratory, Birmingham Women's HospitalBirmingham, United Kingdom; 7 Medical Genetics Branch, National Human Genome Research Institute, National Institutes of HealthBethesda, Maryland; 8 Emory Children's Center Division of Gastroenterology, Hepatology, and NutritionAtlanta, Georgia; 9 Division of Metabolic Disorders, Children's Hospital of Orange CountyOrange, California; 10 Service d'Hépatologie Pédiatrique, CHU BicêtreParis, France; 11 Clinical Genetic Unit, Hôpital Robert DebréParis, France; 12 Neonatology Unit, Mayis UniversitySamsun, Turkey; 13 Bristol Royal Hospital for Sick ChildrenBristol, United Kingdom; 14 Division of Metabolism, Bambino Gesú Children's Hospital IRCCSRome, Italy; 15 Erasmus University Medical Center, Sophia Children's HospitalRotterdam, the Netherlands; 16 Department of Pediatrics, Instituto da Criança, University of Sao PauloSao Paulo, Brazil; 17 Institute of Liver Studies/Histopathology, King's College HospitalLondon, United Kingdom; 18 The Platelet Group, School of Clinical and Experimental Medicine, College of Medical and Dental Sciences, University of BirminghamBirmingham, United Kingdom; 19 Inherited Metabolic Diseases, Great Ormond Street HospitalLondon, United Kingdom

**Keywords:** ostopenia, cholestasis, HOPS complex, recycling endosomes, *VPS33B*, *VIPAR*

## Abstract

Arthrogryposis–renal dysfunction–cholestasis (ARC) syndrome is a rare autosomal recessive multisystem disorder caused by mutations in vacuolar protein sorting 33 homologue B (*VPS33B*) and VPS33B interacting protein, apical–basolateral polarity regulator (*VIPAR*). Cardinal features of ARC include congenital joint contractures, renal tubular dysfunction, cholestasis, severe failure to thrive, ichthyosis, and a defect in platelet alpha-granule biogenesis. Most patients with ARC do not survive past the first year of life. We report two patients presenting with a mild ARC phenotype, now 5.5 and 3.5 years old. Both patients were compound heterozygotes with the novel *VPS33B* donor splice-site mutation c.1225+5G>C in common. Immunoblotting and complementary DNA analysis suggest expression of a shorter VPS33B transcript, and cell-based assays show that c.1225+5G>C VPS33B mutant retains some ability to interact with VIPAR (and thus partial wild-type function). This study provides the first evidence of genotype–phenotype correlation in ARC and suggests that *VPS33B* c.1225+5G>C mutation predicts a mild ARC phenotype. We have established an interactive online database for ARC (https://grenada.lumc.nl/LOVD2/ARC) comprising all known variants in *VPS33B* and *VIPAR*. Also included in the database are 15 novel pathogenic variants in *VPS33B* and five in *VIPAR*. Hum Mutat 33:1656–1664, 2012. © 2012 Wiley Periodicals, Inc.

## Introduction

Arthrogryposis, renal dysfunction, and cholestasis (ARC) syndrome (MIM# 208085) is an autosomal recessive multisystem disorder caused by mutations in vacuolar protein sorting 33 homologue B (*VPS33B*; MIM# 608552) and VPS33B interacting protein, apical–basolateral polarity regulator (*VIPAR*; MIM# 613401) [Cullinane et al., 2010; Gissen et al., 2004]. Characteristic presentation of ARC syndrome includes neonatal cholestatic jaundice, renal tubular acidosis, arthrogryposis, and severe failure to thrive. Most patients fail to survive past the first year of life [Gissen et al., 2006].

VPS33B is a member of the Sec1/Munc18 family proteins, which directly interact with soluble NSF attachment protein receptors (SNAREs) and aid SNARE complex formation, thus facilitating membrane tethering and fusion [Dulubova et al., 2003; Hata et al., 1993; Yamaguchi et al., 2002]. VPS33B and VIPAR are homologues of yeast Vps33 and Vps16, respectively. In yeast, these proteins along with two other class C vps proteins (Vps11 and Vps18) make up the core of homotypic protein sorting (HOPS) and class C core vacuole/endosome tethering (CORVET) complexes responsible for the maturation and control of vesicular trafficking from early to late endosomes and vacuoles [Peplowska et al., 2007]. In addition to four class C vps proteins, HOPS and CORVET complexes contain two other compartment-specific subunits. HOPS contains Vps39 and Vps41, with Vps41 shown to directly interact with Ypt-7-GTP, a Rab7 orthologue [Brett et al., 2008], whereas CORVET contains Vps3 and Vps8. Intermediate complexes containing both CORVET- and HOPS-specific subunits raise the possibility that these complexes may convert “on-the-fly” from one complex to another [Peplowska et al., 2007]. Study of the subunit organization of HOPS and CORVET showed that Vps16 is required for Vps33 association with Vps11 and Vps18 [Rieder and Emr, 1997]; more specifically, residues 451–595 at the C-terminal domain in yeast Vps16 mediates binding to Vps33 [Pulipparacharuvil et al., 2005]. Vps18 has a crucial role in the assembly of HOPS and the removal of Vps18 prevents the coimmunoprecipitation of all other subunits with Vps11 [Plemel et al., 2011]. Mammalian equivalents of HOPS have been studied by several groups [Kim et al., 2001; Nickerson et al., 2009; Zhu et al., 2009]. In metazoans, there are two homologues of yeast Vps33, VPS33A and VPS33B. It is probable that VPS33A in metazoans is a part of the HOPS complex [Sriram et al., 2003], but the conditions in which the VPS33B–VIPAR complex may be involved in HOPS are unknown [Cullinane et al., 2010; Zlatic et al., 2011]. At present, whether there is a mammalian equivalent of the yeast CORVET complex is still unclear [Zlatic et al., 2011].

It has been reported that VPS33B forms a complex with VIPAR and that together they partially colocalize and coimmunoprecipitate with Rab11a. Rab11a is a small GTPase associated with apical recycling endosomes, thus implicating a role for the VPS33B–VIPAR complex in the apical recycling pathway [Cullinane et al., 2010]. Furthermore, mislocalization of apical membrane proteins in the liver and kidneys of ARC patients [Cullinane et al., 2010; Gissen et al., 2004] and structural and functional abnormalities in the apical junction complex in mIMCD3 cells with VPS33B and VIPAR stable knockdown support a role for VPS33B–VIPAR complex in the maintenance of apical–basolateral polarity [Cullinane et al., 2010].

How the VPS33B–VIPAR complex regulates apical basolateral polarity has not been delineated. Identification of disease-causing mutations that modify the structure of wild-type protein can advance understanding of protein interactions and ultimately of their roles in intracellular processes. Thus, the aim of our work was to identify possible links between patient phenotype, genotype, and the resultant effects of mutations on VPS33B–VIPAR localization and interaction with HOPS complex. For the first time, we present two patients with an attenuated ARC phenotype, who both are compound heterozygotes for a novel c.1225+5G>C mutation in *VPS33B*. We summarize all published variants found in *VPS33B* and *VIPAR* to date in a Leiden Open-Source Variation Database (LOVD) for ARC and report 15 novel mutations in *VPS33B* and five in *VIPAR*. Using a cell-based assay, we investigated the effect of various mutations that occur in ARC patients on VPS33B–VIPAR complex formation. In addition, we studied the interaction of the wild-type and mutant proteins with the HOPS complex protein VPS18 to detect any possible functional evidence of the interaction of VPS33B–VIPAR and HOPS complexes.

## Materials and Methods

### Patients and Controls

Patient clinical data have been obtained in a manner conforming with granting agency ethical guidelines. Informed consent was obtained from all participating families; research ethics committees from all participating institutions approved the study. Primary skin fibroblasts from patients were cultured in Dulbecco's modified Eagle's medium (DMEM) supplemented with 10% fetal calf serum, 2 mM L-glutamine, MEM nonessential amino acid solution (all from Sigma–Aldrich, Poole, United Kingdom). DNA was extracted from blood or fibroblasts using the DNeasy blood and tissue kit (Qiagen, Crawley, United Kingdom) according to manufacturer's instructions.

### *VPS33B* and *VIPAR* Sequencing

*VPS33B* and *VIPAR* exons and flanking intronic DNA were sequenced using polymerase chain reaction (PCR) conditions and primers previously described [Cullinane et al., 2010; Gissen et al., 2004]. When exons could not be amplified due to indels, amplification of these regions was carried out by long range PCR, primers were designed within the flanking introns and nested sequencing was performed. Variants were named according to Human Genome Variation Society nomenclature guidelines (+1 as the A of the ATG initiation codon; http://www.HGVS.org) and numbered using the VPS33B reference sequence (NG_012162.1, NM_018668.3) and the VIPAR reference sequence (NG_023421.1, NM_022067.3).

### ARC–LOVD Database

An online locus-specific ARC database (https://grenada.lumc.nl/LOVD2/ARC) was compiled using the LOVD software system [Fokkema et al., 2011]. To establish the database, all relevant data from Human Gene Mutation Database (www.hgmd.org) and sequence variants obtained by literature search for “ARC,” “VPS33B,” and “VIPAR” were collated. The database also contains variants taken from single-nucleotide polymorphism database (http://www.ncbi.nlm.nih.gov/projects/dbSNP) [Sherry et al., 2001]. Any mutations found in patients referred for diagnostic analysis were also included in the database. Detailed description of database construction can be found in Supp. Methods.

### Protein Structure Predictions of VPS33B and VIPAR

Predictions of protein secondary structure, globularity, and disorder were performed using GlobPlot (http://globplot.embl.de/), FoldIndex (http://bip.weizmann.ac.il/fldbin/findex), IUPred (http://iupred.enzim.hu/), RONN (http://www.oppf.ox.ac.uk/RONN/), and HHPRED (http://toolkit.tuebingen.mpg.de/hhpred).

### Complementary DNA Constructs

Complementary DNA (cDNA) constructs of human full-length VPS33B and VPS33B–L30P in the pEYFP-C3 vector and VIPAR in the pCMV-myc vector were used [Cullinane et al., 2010]. A VIPAR–L213P construct was created using the site-directed mutagenesis kit (Stratagene, Stockport, United Kingdom) according to supplied protocol and was sequence verified. The patient AB VPS33B–c.1225+5G>C construct required integration of patient cDNA into the wild-type construct and removal of missing exons.

### Cell Culture and Transfection

All tissue culture reagents were from Sigma–Aldrich unless otherwise stated. HEK293 cells were maintained in high-glucose (4.5 g/l) DMEM medium supplemented with 10% Fetal Bovine Serum (PAA Laboratories, Somerset, United Kingdom), 2 mM L-glutamine, and MEM nonessential amino acid solution. For experiments, HEK293 cells were seeded either on plastic plates or glass coverslips, grown for 24 hr and transfected with plasmid DNA using Lipofectamine 2000 according to manufacturer's protocols (Invitrogen, Paisley, United Kingdom).

### Immunofluorescence Confocal Microscopy

HEK293 cells grown on glass coverslips transfected as above were allowed 24 hr recovery before fixation (4% paraformaldehyde [PFA] in PBS) and permeabilization (0.1% Triton X-100 in PBS). Myc-tagged protein was immunostained with the mouse monoclonal antibody anti-myc (Clone 9E10) (Sigma, Poole, United Kingdom) at a 1:400 concentration and anti-mouse ALEXA-568 conjugate secondary antibody (Invitrogen) at a concentration of 1:400. Nuclei were stained with TO-PRO-3 (Invitrogen). Microscopic images were captured using an inverted Leica TCS SP2 AOBS confocal microscope with a ×63 oil immersion objective (N/A 1.4) and 3× optical zoom; the pinhole was set to 1 Airy unit. A series of optical sections were collected from *xy* plane and merged into maximum projection images. Figures were prepared using Photoshop.

### Immunoelectron Microscopy

HEK293T cells were fixed by adding freshly prepared 4% PFA or 4% PFA + 0.4% glutaraldehyde (GA) (w/v) (Polysciences, Eppleheim, Germany) in 0.1 M phosphate buffer (pH 7.4) to an equal volume of culture medium for 10 min, followed by postfixation in 4% PFA or 2% PFA + 0.2% GA (w/v) at 4°C overnight. Ultrathin cryosectioning and immunogold labeling were performed as described [Slot and Geuze, 2007]. Fixed cells were washed with PBS containing 0.05 M glycine, scraped gently free, and embedded in 12% gelatin in PBS. The cell pellet was solidified on ice and cut into small blocks. For cryoprotection, blocks were infiltrated overnight with 2.3 M sucrose at 4°C and then mounted on pins and frozen in liquid nitrogen. Ultrathin cryosections at 70 nm were prepared on a Leica ultracut UCT ultra cryomicrotome and picked up with a freshly prepared 1:1 mixture of 2.3 M sucrose and 1.8% methylcellulose [Liou et al., 1996]. Ultrathin cryosections were then immunogold labeled and examined using a JEOL TEM 1010 electron microscope at 80 kV.

Antibodies used included biotinylated polyclonal goat anti-GFP (Rockland, Gilbertsville, PA) used to localize YFP-tagged proteins; polyclonal rabbit anti-biotin (Rockland) was used as a bridging antibody between the biotinylated anti-GFP antibody and the protein A-gold, monoclonal anti-myc (9E10) (Santa Cruz Biotechnology, Heidelberg, Germany), monoclonal anti-Tf receptor (Zymed, Barcelona, Spain), and a polyclonal rabbit anti-mouse antibody (DAKO, Glostrup, Denmark) to bridge mouse monoclonal antibody to protein A-gold.

## Results

### Identification of Patients with an Attenuated ARC Phenotype

**Figure 1:**
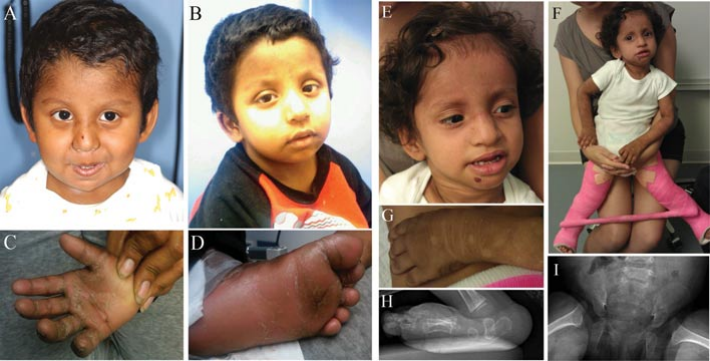
Patients with attenuated ARC syndrome. **A–D:** Patient AB. Aged (**A**) 3 years and (**B**) 5 years. Hyperkeratosis and lichenification of palm of right hand (**C**) and sole of left foot (**D**). **E–I:** Patient CD. Facies aged 3 (**E**); in plaster after corrective hip surgery (**F**). Hyperkeratosis, dorsum of right foot (**G**). Radiographs of right foot showing vertical talus (**H**) and of pelvis showing hip dislocation (**I**).

Patients with the suspected diagnosis of ARC were referred to our group for clinical and molecular diagnosis. Twenty novel mutations in *VPS33B* and *VIPAR* were identified in patients with the classical features of ARC (see Table 1). Patient AB was referred for mutation screening at 2.5 years with what appeared to be an attenuated phenotype of ARC. AB is of nonconsanguineous Peruvian and Puerto Rican descent. His clinical features included failure to thrive, developmental delay with sensorineural hearing loss, renal loss of protein and amino acids, bilateral talipes with osteopenia, and mild cholestasis. Magnetic resonance imaging (MRI) showed dysmorphic ventricles with coaptation of the occipital horns and irregular lateral–ventricular marginal contours. Mild confluent hyperintense T2 signal in the periventricular white matter suggested early in utero ischemic injury. Troublesome pruritus and ichthyosis were associated with increased serum concentrations of bile acids and did not respond to conventional therapy. Clinical management with supplemental feeds via gastric tube achieved steady growth along the 0.3rd percentile for his weight and height. Pruritus responded to cutaneous biliary diversion performed at age 3 years (Fig. 1A–D).

**Table 1:** Novel Pathogenic *VPS33B* and *VIPAR* Variants Listed in ARC Database

Database ID	Gene	Exon	DNA Change	Protein Change
VPS33B_00221	*VPS33B*	1	c.67C>T	p.(Arg23*)
VPS33B_00223	*VPS33B*	1i	c.97-2A>C	p.(?)^a^
VPS33B_00231	*VPS33B*	2i	c.178-2A>C	p.(?)
VPS33B_00224	*VPS33B*	2i	c.178-1G>C	p.(?)
VPS33B_00225	*VPS33B*	10	c.711del	p.(Phe237Leufs*2)
VPS33B_00226	*VPS33B*	11i	c.853-3C>G	p.(?)
VPS33B_00227	*VPS33B*	13i	c.1030+5G>T	p.(?)
VPS33B_00230	*VPS33B*	16i	c.1225+5G>C	p.(?)
VPS33B_00229	*VPS33B*	17	c.1261_1262del	p.(Gln421Valfs*8)
VPS33B_00228	*VPS33B*	20	c.1498G>T	p.(Glu500*)
VPS33B_00232	*VPS33B*	Δ4	c.240-577_290-156del	p.(Leu81Serfs*5)
VPS33B_00233	*VPS33B*	3i	c.240-1G>C	p.(?)
VPS33B_00234	*VPS33B*	21i	c.1657+1G>A	p.(?)
VPS33B_00226	*VPS33B*	11i	c.853-3C>G	p.(?)
VPS33B_00235	*VPS33B*	1-23	c.(?_-354)_(*431+d127_?)del	p.(0?)^b^
VIPAR_00023	*VIPAR*	9	c.638T>C	p.(Leu213Pro)
VIPAR_00021	*VIPAR*	6	c.463_464del	p.(Trp155Glufs*4)
VIPAR_00022	*VIPAR*	6	c.484C>T	p.(Arg162*)
VIPAR_00019	*VIPAR*	13	c.1021T>C	p.(Cys341Arg)
VIPAR_00020	*VIPAR*	11i	c.837-1G>T	p.(?)

Variants were numbered using the *VPS33B* RefSeq (NG_012162.1, NM_018668.3) and *VIPAR* RefSeq (NG_023421.1, NM_022067.3).

Del, deletion; Fs, frameshift; i, intron; *, stop; Δ, whole exon deletion.

a p.(?) effect of the variant upon the protein is unknown.

b p.(0?) no protein product is predicted.

Sequencing of *VPS33B* revealed that Patient AB is a compound heterozygote for mutations in *VPS33B*. One allele harbors the deletion c.240–577_290-156del; absence of exon 4 from the cDNA results in a frameshift and premature stop codon, p.(Leu81Serfs*5) (see Supp. Fig. S1). The other allele harbors the donor splice-site mutation c.1225+5G>C.

At the age of 5.5 years, AB attends kindergarten and a special school for children with hearing impairment. He is making slow progress in his development, such as learning to say two-syllable words. AB has difficulties with using sign language due to severe hyperkeratosis and lichenification of the skin of his hands. He can walk unaided. He likes to play with other children particularly basketball, when he likes to dribble the ball and to shoot baskets. He can ride a bicycle with training wheels. Although thick, calloused hand skin (Fig. 1C) interferes with fine motor tasks, this problem seems to be responding to dermatological management.

AB continues to have osteopenia with shortening of the proximal fibula, generalized aminoaciduria and nephrotic range proteinuria, and recurrent episodes of epistaxis associated with the absence of platelet alpha-granules.

Patient CD, with the features of possible ARC, was referred for a clinical assessment aged 12 months. Her parents are of Puerto Rican and Jualisco Mexican origin. She was found to have arthrogryposis and failure to thrive at age of 2 weeks. Investigations identified renal tubular dysfunction, mild cholestasis, hyperpigmented lichenified skin, bilateral hip dislocations, decreased muscle bulk, and sensorineural hearing loss (Fig. 1E–I). She was not jaundiced but had hypercholanemia. MRI at 14 months showed a thin corpus callosum and diffuse paucity of white matter. However, she continued to progress in development with appropriate socialization. Speech improved after a hearing aid was fitted. Pruritus improved with rifampicin, phenobarbitone, and ursodeoxycholic acid treatment. CD continues to fail to thrive despite supplementary feeds. At age 3 years, she underwent corrective surgery for hip dysplasia; she is starting to walk. Additional clinical problems include abnormal dental composition with weak enamel and easily chipped teeth (Fig. 1E).

Sequencing by Prevention Genetics found the patient to be a compound heterozygote for mutation in *VPS33B*. One allele harbors the mutation c.1261_1262delCA, predicted to result in a frameshift and premature protein termination (p.Gln421Valfs*8). The other harbors the mutation c.1225+5G>C, the splice donor site mutation identified in patient AB.

Although the mutations present were predicted to yield a severe phenotype, both AB and CD had an attenuated form of ARC. This finding merited further investigation. Immunoblotting of protein obtained from AB's fibroblasts indicated the presence of VPS33B protein product shorter than control (Supp. xFig. S2C). When 3′-RACE was used to amplify the *VPS33B* transcript of unknown composition resulting from the splice-site mutation, 114 bp of intron 16 were found integrated into the *VPS33B* transcript before termination with a polyA tail (Supp Fig. S2B). The predicted protein composition therefore includes 12 additional amino acid residues, encoded by this intronic sequence, before a stop codon. Exons 17–23 are absent from the cDNA, thus resulting in a predicted protein length of 420 aa in comparison to the wild-type 617aa (Supp. Fig. S2D).

### Mutations in *VPS33B* and *VIPAR* and ARC–LOVD Database

We have compiled an online locus-specific ARC database (https://grenada.lumc.nl/LOVD2/ARC), using the LOVD software system [Fokkema et al., 2011] that lists all identified variants in *VPS33B* and *VIPAR*. The database includes 15 previously unpublished variants in *VPS33B* and five in *VIPAR* that have been classed as “pathogenic” (Table 1).

**Figure 2:**
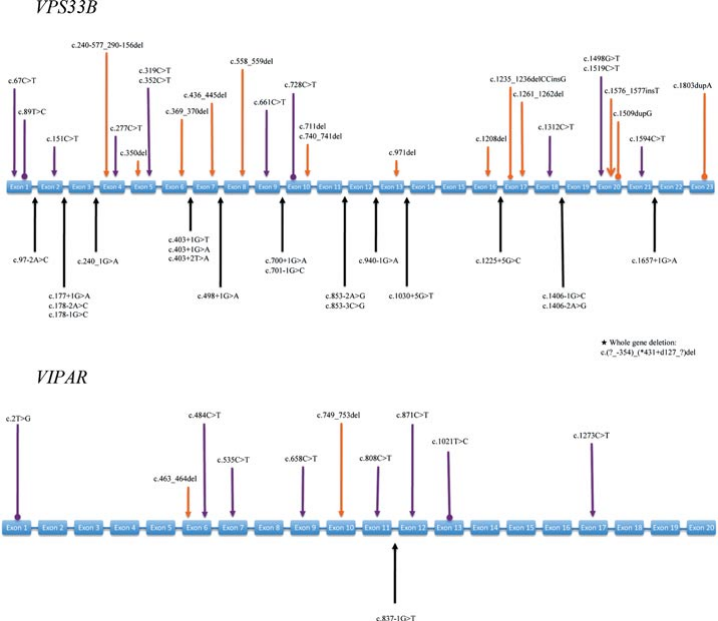
ARC–LOVD database content analysis. ***VPS33B*** Gene map of distribution of “pathogenic” and “probably pathogenic” variants in *VPS33B*. The boxes represent the exons (not to scale). All variants are described at the DNA level. Variants below the genogram represent intronic mutations, affecting splice sites. Variants above the genogram represent mutations in exons. Purple pointers indicate substitutions; → indicates a nonsense mutation, —• indicates a missense mutation. Orange pointers indicate deletions/duplications/indels; → indicates a deletion, —• indicates a duplication, and —♦ indicates an indel. ***VIPAR*** Gene map of distribution of “pathogenic” and “probably pathogenic” variants in *VIPAR*. The boxes represent the exons (not to scale). All variants are described at the DNA level. Variants above the genogram represent mutations in the exons. Purple pointers indicate substitutions; → indicates a nonsense mutation, —• indicates a missense mutation. The orange pointer → indicates a deletion.

As of January 2012, the ARC database contained 235 unique variants in *VPS33B* and 23 in *VIPAR*. Forty-seven variants in *VPS33B* have been classed as “pathogenic” or “probably pathogenic” due to their predicted effect on the protein and the clinical presentation of the patients in whom they were found (Fig. 2). Most of these are substitutions (*n* = 32; 20 splice site, 10 nonsense, and two missense). There are also deletions (*n* = 9; including 1 whole gene deletion), duplications (*n* = 2), insertions (*n* = 1), and indels (*n* = 1), all of which are predicted to result in frameshift and premature termination of transcription.

There are nine recurrent variants in *VPS33B*, of which three are more prevalent; a splice-site mutation, c.403+2T>A (reported in 11 individual patients, all of Korean origin), a nonsense mutation, c.1312C>T (reported in nine patients; eight of Pakistani origin, and one Arabic), and a nonsense mutation c.1519C>T (reported in seven patients; three Portuguese, one Korean, one Hispanic, two unknown ethnicity) (references within the database). Molecular diagnosis in research and service laboratories in addition has identified c.1312C>T and c.1519C>T mutations in additional patients with ARC from relevant ethnic groups (Paul Gissen, personal communication).

Of 11 “pathogenic” variants in *VIPAR* (Fig. 2), most are substitutions (*n* = 8; five nonsense and two missense), with two deletions (resulting in frameshift) and one splice-site mutation. Two variants recur: the nonsense mutation, c.658C>T (reported in two patients; ethnic origins Italian and Turkish) and the nonsense mutation, c.808C>T (reported in two patients; ethnic origins Pakistani and Arab–Israeli).

The distribution of variants is relatively uniform within VPS33B; we identified no obvious mutational hotspots (Fig. 2). Protein structure predictions for VPS33B and VIPAR suggest that both proteins contain globular regions with well-defined secondary structure elements. VPS33B contains a Sec1 domain (amino acids 31–611) spanning almost the entire protein. Structure–prediction software has consistently identified a large disordered protein segment at the N-terminus of VIPAR. At present, with the exception of a single pathogenic mutation in the start codon, the N-terminal part of VIPAR is devoid of mutations. This might suggest that this region of VIPAR is dispensable for VPS33B–VIPAR interaction. Protein secondary structure, globularity, and disorder were predicted using GlobPlot (http://globplot.embl.de/), FoldIndex (http://bip.weizmann.ac.il/fldbin/findex), IUPred (http://iupred.enzim.hu/), RONN (http://www.oppf.ox.ac.uk/RONN/), and HHPRED (http://toolkit.tuebingen.mpg.de/hhpred).

### Identification of a Correlation between Genotype and Cellular Phenotype

Nonsense and frameshift mutations in *VPS33B* and *VIPAR* are associated with severe clinical phenotype and likely result in absent protein product due to premature termination of transcription and nonsense-mediated decay [Cullinane et al., 2009]. The missense mutations identified so far likely result in an expressed protein product, however are also associated with severe clinical phenotype. Finally, a novel splice-site mutation in *VPS33B* reported here, c.1225+5G>C, resulting in a truncated protein product, is associated with an attenuated ARC phenotype.

Mutations predicted to result in an expressed protein from patients with phenotypes assessed as severe (*VPS33B* p.Leu30Pro and *VIPAR* p.Leu213Pro) and moderate (*VPS33B* c.1225+5G>C) were selected from the database for modeling to gain further insight into disease pathogenesis and VPS33B and VIPAR function (Supp. Table S1 ).

Overexpression of wild-type VPS33B and VIPAR within HEK293 cells has found that these two proteins strongly colocalize to defined spots, and that these partially colocalize with RAB11A, a marker for recycling endosomes [Cullinane et al., 2010].

**Figure 3:**
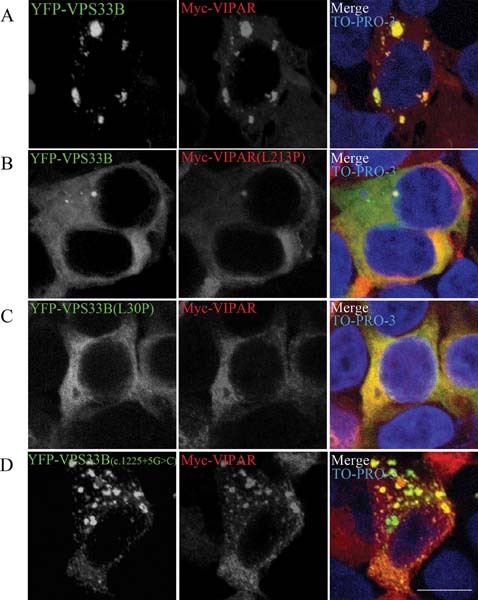
VPS33B and VIPAR interaction. Confocal fluorescence photomicrographs of HEK293 cells cotransfected with wild-type and mutant constructs of YFP-tagged VPS33B and Myc-tagged VIPAR. Wild-type YFP-VPS33B and Myc-VIPAR colocalized (**A**). However, constructs modeled on severe phenotype mutant proteins (**B**) Myc-VIPAR(L213P) and (**C**) YFP-VPS33B(L30P) resulted in a disruption of VPS33B–VIPAR interaction. **D:** Transfection for YFP-VPS33B(c.1225G>C), modeling an attenuated phenotype, resulted in partial colocalization. Myc-VIPAR was immunostained with mouse monoclonal antibody anti-myc (Sigma) at a 1:400 concentration and anti-mouse ALEXA-568 conjugate secondary antibody (Invitrogen) at a concentration of 1:400. Nuclei are stained with TO-PRO-3. Scale bars, 10 μm.

We used transfections of epitope-tagged constructs to investigate whether patient mutations in VPS33B or VIPAR disrupt the interaction between these proteins when overexpressed in HEK293 cells using colocalization studies. Although wild-type VPS33B and VIPAR colocalized (Fig. 3A), co-overexpression of VPS33B and VIPAR(L213P) mutant did not result in colocalization; a cytoplasmic distribution for both proteins was observed (Fig. 3B). Similarly, co-overexpression of VPS33B(L30P) mutant with VIPAR (Fig. 3C) also resulted in cytoplasmic distribution for both proteins. The severe clinical phenotype associated with these mutations confirms the importance of VPS33B–VIPAR complex formation for the function of these proteins. Finally, when VPS33B(c.1225+5G>C) mutant (known to be associated with an attenuated clinical phenotype, see above) was co-overexpressed with wild-type VIPAR (Fig. 3D), specific fluorescent spots containing both proteins were observed. In addition, aggregates containing only VPS33B could be seen. Thus, it appears that the c.1225+5G>C VPS33B mutant retains partial ability to interact with VIPAR.

**Figure 4:**
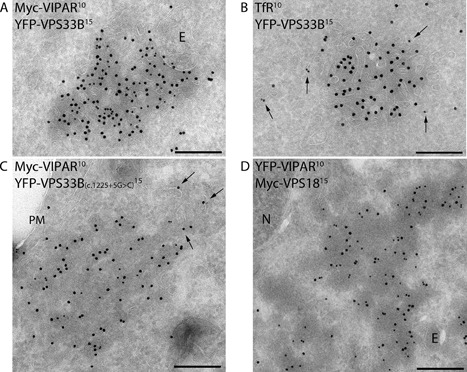
Ultrastructual localizations of VPS33B, VPS33B(c.1225G>C), VIPAR, and VPS18 constructs. Transmission electron micrographs of ultrathin cryosections of HEK293 cells. **A, B:** Cells cotransfected with YFP-tagged VPS33B and Myc-tagged VIPAR were immunogold stained with anti-GFP/YFP (15 nanometer gold) and anti-Myc (10 nanometer gold) (**A**), or anti-GFP/YFP (15 nanometer gold) and anti-TfR (10 nanometer gold) (**B**). Colocalization was observed on endosome (E)-associated tubular–vesicular membranes typical of recycling endosomes, which was confirmed by the presence of TfR (arrows in B). (**C**) Cells co-overexpressing YFP-VPS33B(c.1225G>C) and Myc-VIPAR were immunogold labeled for anti-GFP/YFP (15 nanometer gold) and Myc (10 nanometer gold). The two proteins colocalized in cytosolic aggregates with partial staining of VPS33B(c.1225G>C) on nearby vesicles (arrows). **D:** Cells co-overexpressing YFP-tagged VIPAR (labeled with anti-GFP; 10 nanometer gold) and Myc-tagged VPS18 (labeled with anti-Myc, 15 nanometer gold) showed colocalization in cytosolic aggregates. E, endosome; N, nucleus; PM, plasma membrane. Scale bars, 200 nm.

To investigate the sites of colocalization of VPS33B and VIPAR in ultrastructural detail, we performed cryo-immunogold electron microscopy (EM) of HEK293 cells overexpressing different combinations of constructs. This showed that the wild-type proteins colocalized on endosome-associated tubular–vesicular membranes also positive for transferrin receptor (Fig. 4A and B). The morphological and molecular characteristics of these membranes, including the presence of Rab11A as seen by immunofluorescence, identifies them as recycling endosomes [Sachse et al, 2002]. When VPS33B (c.1225+5G>C) mutant was cotransfected with VIPAR, the two proteins colocalized mainly in electrondense cytoplasmic clusters that contained no membranes (Fig. 4C). Interestingly however, some staining for VPS33B(c.1225+5G>C) could be detected on vesicles found at the rims of these cytoplasmic aggregates. Thus, the VPS33B(c.1225+5G>C) mutation seems to affect the ability of the complexes to colocalize properly on tubular–vesicular recycling membranes, which is likely to abrogate at least partially its cellular functioning.

To identify whether VPS33B and/or VIPAR may interact with the mammalian HOPS complex, we studied their colocalization with human VPS18 protein, which has a central role in HOPS subunit interactions [Ostrowicz et al., 2010; Plemel et al., 2011].

**Figure 5:**
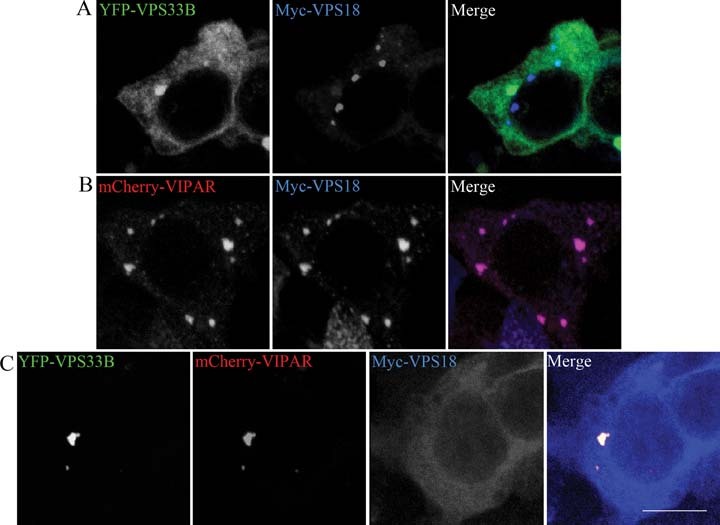
VPS33B and VIPAR interaction with VPS18. Confocal fluorescence photomicrographs of HEK293 cells cotransfected with (**A**) YFP-VPS33B and Myc-VPS18 found these proteins do not colocalize. However, (**B**) mCherry–VIPAR and Myc-VPS18 do colocalize in clusters in the absence of YFP-VPS33B. If all three proteins were cotransfected, then YFP-VPS33B and mCherry-VIPAR formed clusters and Myc-VPS18 assumed independent cytoplasmic distribution (**C**). Myc-VPS18 was immunostained with mouse monoclonal antibody anti-myc (Sigma) and anti-mouse ALEXA-568 conjugate secondary antibody (Invitrogen), both at a concentration of 1:400. Scale bar, 10 μm.

When VPS18 was co-overexpressed with VPS33B, the two proteins did not colocalize: VPS33B was cytoplasmic and VPS18 formed small spots (Fig. 5A). When VPS18 was co-overexpressed with VIPAR, there was colocalization in large fluorescent spots (Fig. 5B). However, by immuno-EM these spots were identified as cytoplasmic aggregates, without any membranes associated (Fig. 4D).

Interestingly, when all three constructs were cotransfected into HEK293 cells (Fig. 5C), VIPAR colocalized and formed clusters with VPS33B, whereas VPS18 was cytoplasmic. These interactions were confirmed by coimmunoprecipitation experiments: HA-VIPAR could pull down members of the HOPS complex, especially VPS18 where a strong interaction was seen (Supp. Fig. S3A). However, when this experiment was repeated in the presence of overexpressed YFP-VPS33B, this strong interaction diminished considerably, confirming the immunofluorescence findings (Supp. Fig. S3B).

## Discussion

ARC is a severe multisystem childhood disorder caused by mutations in either *VPS33B* or *VIPAR*. Here, we report 20 novel mutations in *VPS33B* or *VIPAR*. Also, for the first time we describe two patients with an attenuated ARC phenotype. They have in common the c.1225+5G>C splice-site mutation in *VPS33B*, which we have shown to result in a truncated protein that may retain some wild-type function as a result of forming VPS33B–VIPAR complex-containing clusters. In contrast, the mutated proteins VPS33B(L30P) and VIPAR(L213P), which are associated with a severe ARC phenotype, did not form clusters with their respective wild-type partners. The mutation L30P, modeled using bioinformatics analysis [Gissen et al., 2005], is predicted to disrupt an alpha-helix. Predicted secondary structure of wild-type VIPAR includes an extended disordered region at the N-terminus (residues 1–150), followed by a 100% alpha-helical domain that extends to the C-terminus (residues 105–491). According to these predictions, residues 209–218 are part of a buried alpha-helical segment. A Leu213Pro substitution in VIPAR is predicted to result in the disruption of this alpha-helix in the core of a folded region of VIPAR structure, and might affect the overall folding of the protein and its interactions with VPS33B and/or other potential binding partners.

A splice site c.1225+5G>C mutation in *VPS33B* generates a truncated form of VPS33B (residues 1–420) that still partially colocalizes with VIPAR (Fig. 3D). From this evidence and also the EM experiments, we can affirm that truncation of the C-terminal region of VPS33B, as seen with the c.1225+5G>C splice mutation, may alter the overall interaction between VPS33B and VIPAR and the localization of the complex. However, the attenuated clinical phenotype and the presence in cell culture of VPS33B- and VIPAR-containing clusters suggest that some of the function of the VPS33B–VIPAR complex may be retained. Point mutations in the same C-terminal region might alter folding throughout VPS33B secondary structure and completely abrogate normal interaction with VIPAR. The finding that clusters containing VPS33B(c1225+5G>C) and VIPAR are mainly cytoplasmic suggests that the expected interaction of SM protein VPS33B with transmembrane SNAREs might be important for the proper localization and function of the complex. In addition, interaction with SNARE proteins might be possible only after VIPAR binding to VPS33B, since overexpression of VPS33B alone is not sufficient to localize VPS33B on recycling endosomes.

We also studied VPS33B and VIPAR interaction with the core HOPS complex protein VPS18. As our results suggest that VIPAR may interact with VPS18 only in the absence of VPS33B, the VPS33B–VIPAR complex is unlikely to be involved in HOPS interaction. However, VIPAR may interact with VPS18 in cells with mutant VPS33B, implying that this interaction might be involved in ARC pathogenesis.

The HOPS complex is involved in biogenesis of late endosomes and phagosomes and in cargo delivery to the vacuole/lysosome, thus resulting in protein degradation in both yeast and multicellular organisms [Lindmo et al., 2006; Nickerson et al., 2009; Pulipparacharuvil et al., 2005; Rieder and Emr, 1997; Sevrioukov et al., 1999; Sriram et al., 2003]. We have shown that in cell lines with VPS33B or VIPAR knockdown, degradation of some apical membrane proteins is increased [Cullinane et al., 2010]. Thus, it is conceivable that deficiency of VPS33B or VIPAR in ARC results in rerouting of apical membrane proteins into lysosomes.

Our work broadens the clinical phenotype of VPS33B and VIPAR deficiencies and provides increased understanding of the effect of protein alteration on intracellular trafficking. We have developed the ARC–LOVD database to provide a resource for researchers and clinicians, allowing easy access to a central log of updated information, and to provide the opportunity to share data. We hope that as data are added, opportunities for epidemiological investigations and meta-analysis will increase. The identification of a range of phenotypic severity in ARC underlines the importance of such a database to help accurately counsel patient families on prognosis. In addition, advances in knowledge of ARC pathogenesis and clinical course may lead to novel therapies and improved management of patients with ARC.
