# Supplementary material for: Associations Among Genotype, Clinical Phenotype, and Intracellular Localization of Trafficking Proteins in ARC Syndrome
Source: Hum Mutat. 2012 Jul 2;33(12):1656–64. doi: 10.1002/humu.22155 (PMC3746110; doi:10.1002/humu.22155)
Supplement: Supplementary file 1 [file humu0033-1656-SD1.pdf]

SUPPORTING INFORMATION

Supp. Methods

ARC-LOVD Database Construction

We have compiled an online locus-specific ARC database (https://grenada.lumc.nl/LOVD2/ARC), using the LOVD software system (Fokkema et al., 2011) that lists all identified variants in *VPS33B* and *VIPAR*. Most information is made public and accessible to all users, and the database allows the submission of reports.

To establish the database we added all relevant data from the Human Gene Mutations Database (www.hgmd.org) and performed a literature search. We entered the terms 'VPS33B', 'VIPAR' and 'ARC syndrome' into PubMed (NCBI) and screened abstracts and full-text articles for variants. The database also contains 188 unique variants taken from dbSNP (http://www.ncbi.nlm.nih.gov/projects/SNP) (Sherry et al., 2001) and the most up to date information from the 1000 Genomes Project (http://www.1000genomes.org/), including information on frequency. Patients with classical ARC phenotype were referred for clinical advice and also for molecular diagnosis to the University of Birmingham and West Midlands Regional Genetics laboratories where all coding exons and intron-exon boundaries were screened for mutations in *VPS33B* and *VIPAR*. One patient was screened for mutations in *VPS33B* by Prevention Genetics (http://www.preventiongenetics.com). Here we report 19 previously unpublished pathogenic variants.

Variant data for *VPS33B* and *VIPAR* were initially stored in separate offline Microsoft Excel worksheets (Microsoft, Redmont, WA) and were subsequently imported in tab-delimited format into the ARC database within the LOVD platform (Fokkema et al., 2011). Variants were named

according to HGVS nomenclature guidelines (http://www.HGVS.org) and numbered using the *VPS33B* reference sequence (NG_012162.1, NM_018668.3) and the *VIPAR* reference sequence (NG_023421.1, NM_022067.3). Previously published mutations were renamed accordingly and all annotations were checked using Web-based Mutalyzer software linked to LOVD (Wildeman et al., 2008).

VPS33B and *VIPAR* each have a gene homepage. This provides general information about the gene, as well as a guide to the variant tables in the database and an explanation of the searches that can be implemented. Additional information about the gene is also accessible through links to other resources provided on the homepage. In the "variants" section are variant-specific fields for each gene (exon, pathogenicity, template/technique used for detection, DNA/RNA/protein change, frequency, number of times reported, database ID, phenotype, and reference). Records describing variants per individual patient can be accessed, along with information on patient phenotype and ethnic/geographic origin. The database also contains a "documentation" section, which includes a guide for submitters. After initial registration, new variants can be submitted directly to the database, where they will be made public following curation.

Characterization of new variants in *VPS33B* and *VIPAR*

Of the disease-causing variant types, most common are substitutions (n=11: 9 splice-site and 2 nonsense). The effect of the splicing mutations was predicted by bioinformatic analysis using the BDGP Splice Site Prediction software NNSPLICE (http://www.fruitfly.org/seq_tools/splice.html). We identified 3 deletions which both are predicted to result in frameshift and premature termination of transcription, as well as one whole-

gene deletion. A variant of particular interest is the splicing mutation c.1225+5G>C, as it is associated with an attenuated ARC phenotype.

We also report 5 novel mutations in VIPAR (Table 1), including substitutions (n=3: 2 missense and 1 nonsense), a deletion predicted to result in frameshift and premature termination of transcription, and a splicing mutation. All are classed as ‘pathogenic’ or ‘probably pathogenic’.

qRT-PCR to determine exon copy number

qRT-PCR was performed using 25ng genomic DNA to identify deletions of *VPS33B* exon 4 using the method described (Hoebeeck et al. 2004) Normalization was carried out using 2 reference genes, *GAPDH* and *actin* with normal copy number. qRT-PCR was performed with Power SYBR Green MasterMix (Applied Biosystems, Warrington, UK) and an ABI 7500 thermal cycler (Applied Biosystems, Warrington, UK).

Co-Immunoprecipitation

For co-immunoprecipitation, 20 µg of anti-HA monoclonal antibodies were covalently conjugated to 100 µl of Dynabeads Protein G (Invitrogen, Paisley, UK) using dimethyl pimelimidate and triethanolamine according to the manufacturer’s instructions. HEK293 cells growing on 6-well plates and transfected with a total of 4 **µ**g of plasmid DNA constructs. These were allowed to recover for 48h before the protein was extracted as above. Extracted proteins (250µg) were mixed with 20 µL of antibody-conjugated Dynabeads and incubated on a blood rotor with end-over-end mixing (at 4°C for 3 h). The complexes were then washed 3 times using cell lysis buffer supplemented with 150 mM NaCl, after which proteins were eluted by boiling in

2x SDS loading buffer (10 min). The protein samples were loaded directly onto SDS-PAGE gels for immunoblotting analysis

Protein extraction

Protein was extracted from patient fibroblasts or from HEK293 cells washed with ice cold PBS and scraped into 1ml or 200µl of lysis buffer containing 50 mM Tris-HCl (at pH 7.5), 50 mM sodium fluoride, 5 mM sodium pyrophosphate, 1 mM sodium orthovanadate, 1 mM EDTA, 1 mM EGTA, 0.27 M sucrose, 1% Triton X-100 and Complete, Mini Protease Inhibitor Cocktail (Roche Diagnostics, West Sussex, UK). Cell lysates were centrifuged (15,000 RPM, 15 min at 4 °C) and supernatants were removed for immunoblotting.

Immunoblotting

The absence or presence of VPS33B was determined by separating 25µg of extracted protein on a 12% SDS-PAGE gel. Proteins were transferred to transblot polyvinylidene difluoride membranes (Hybond-P; Amersham Biosciences, Little Chalfont, UK).

Immunoblotting was carried out according to standard protocols (Harlow and Lane, 1998) using a 1/500 dilution of the primary antibody rabbit anti-human VPS33B (Proteintech, Manchester, UK) and a 1/1000 dilution of goat anti-rabbit HRP conjugate (Dako, Cambridge, UK). The membrane was stripped by boiling in water and re-blotted for actin as a loading control using 1/15000 dilution of mouse anti-human β-actin (cloneAC-15) (Sigma, Poole UK) and a 1/20000 dilution of rabbit anti-mouse HRP conjugate (Dako, Cambridge UK). For the co-

immunoprecipitation experiments, immunoblotting for myc and HA used a 1/5000 dilution mouse anti-Myc (clone 9E10) (Sigma-Aldrich, Poole, UK) and 1/10000 dilution of mouse anti-HA (clone HA-7)(Sigma-Aldrich, Poole, UK). For the secondary antibody a 1/5000 dilution of rabbit anti-mouse HRP conjugate (Dako, Cambridge, UK) was used.

RNA Extraction and cDNA Synthesis

Fibroblast cells were grown to confluence in 75 cm^2 flasks and HEK293 cells were grown in 6-well plates before RNA was extracted using RNAzol B reagent (Campro Scientific, UK) according to manufacturer's instructions. For cDNA synthesis, 1μg of total RNA was reverse transcribed using ImProm-II Reverse Transcription Systems and oligo dT primers (Promega, Southampton, UK) according to manufacturer's protocol. 3'-RACE was carried out using the SMART RACE cDNA Amplification kit (Clontech, UK) according to manufacturer's conditions.

Supp. References

Hoebeeck J, van der Luijt R, Poppe B, De Smet E, Yigit N, Claes K, Zewald R, de Jong GJ, De Paepe A, Speleman F, Vandesompele J. 2005. Rapid detection of VHL exon deletions using real-time quantitative PCR. Lab Invest 85: 24-33.

Wildeman M, van OE, den Dunnen JT, Taschner PE. 2008 Improving sequence variant descriptions in mutation databases and literature using the Mutalyzer sequence variation nomenclature checker. Hum Mutat 29: 6-13.

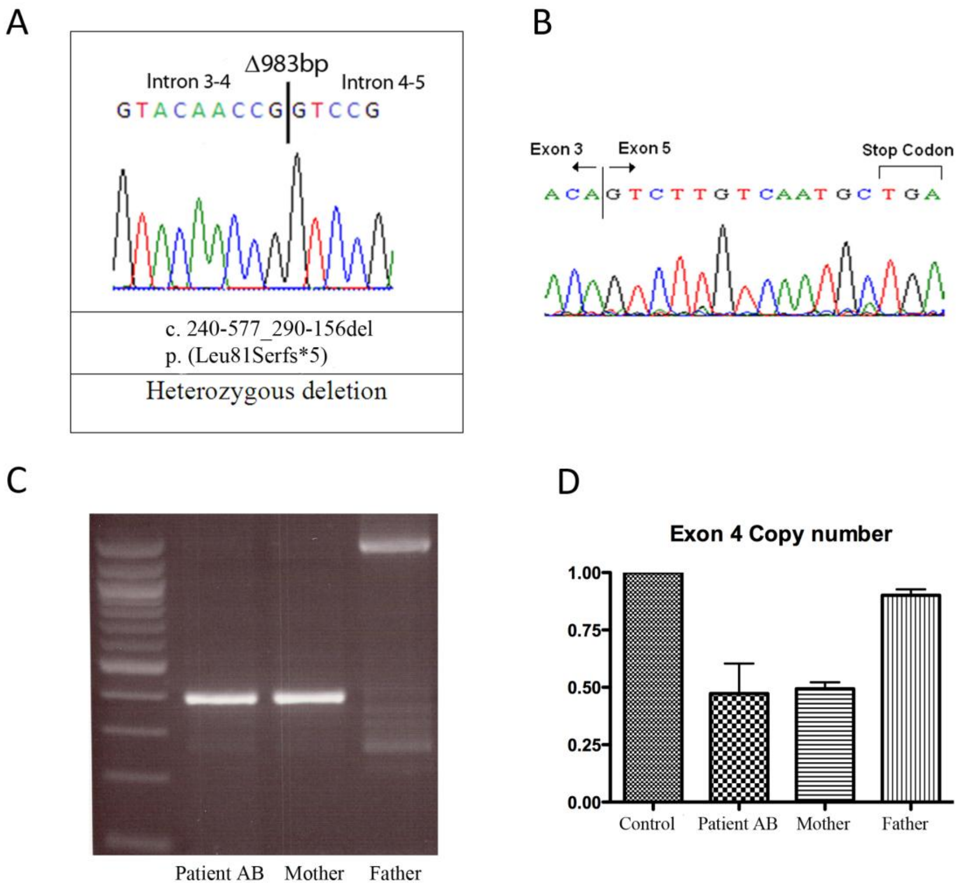

Supp. Figure S1. Characterization of the heterozygous deletion mutation in Patient AB. (A) Electropherogram identifying the location of the 983bp deletion- c.240-277_290-156del in *VPS33B* and an electropherogram (B) of the resultant cDNA lacking exon 4. The deletion results in a frameshift and premature stop codon in *VPS33B*. (C) PCR products using primers flanking the deletion indicate that maternal DNA contains the deletion but DNA from father and control do not. qRT-PCR results using genomic DNA (D) confirm this finding. Exon 4 is normalized against GAPDH and actin. Samples containing 2 copies of the exon will give the result 1, and those containing one copy due to a heterozygous deletion will give the result 0.5. Samples from the mother and Patient AB contain only one copy of exon 4.

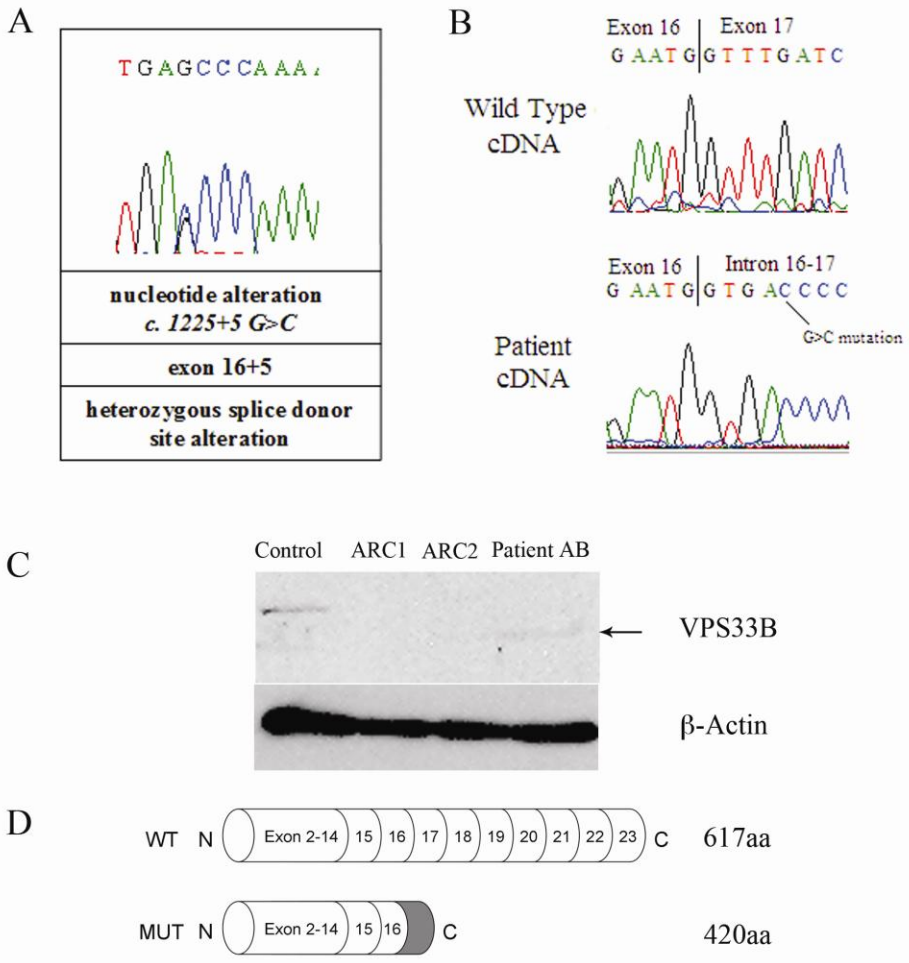

Supp. Figure S2. Characterization of the heterozygous splice site mutation in Patient AB. Electropherograms showing (A) the c.1225+5G>C mutation and (B) the difference in *VPS33B* cDNA composition between wild-type and patient; 114bp of intronic sequence after exon 16 was integrated into the *VPS33B* transcript before termination with a polyA tail. (C) VPS33B immunoblot using protein obtained from control fibroblasts (lane 1), patients with c.1312C>T (lane 2), c.1594C>T and c.1225+5G>C (lane 4) identifies a smaller protein product in lane 4. (D) A diagram showing the predicted protein composition of mutant (MUT) VPS33B in comparison to wild type (WT) including 12 additional amino acid residues encoded by the intronic sequence before a putative stop codon. Exons 17-23 are absent.

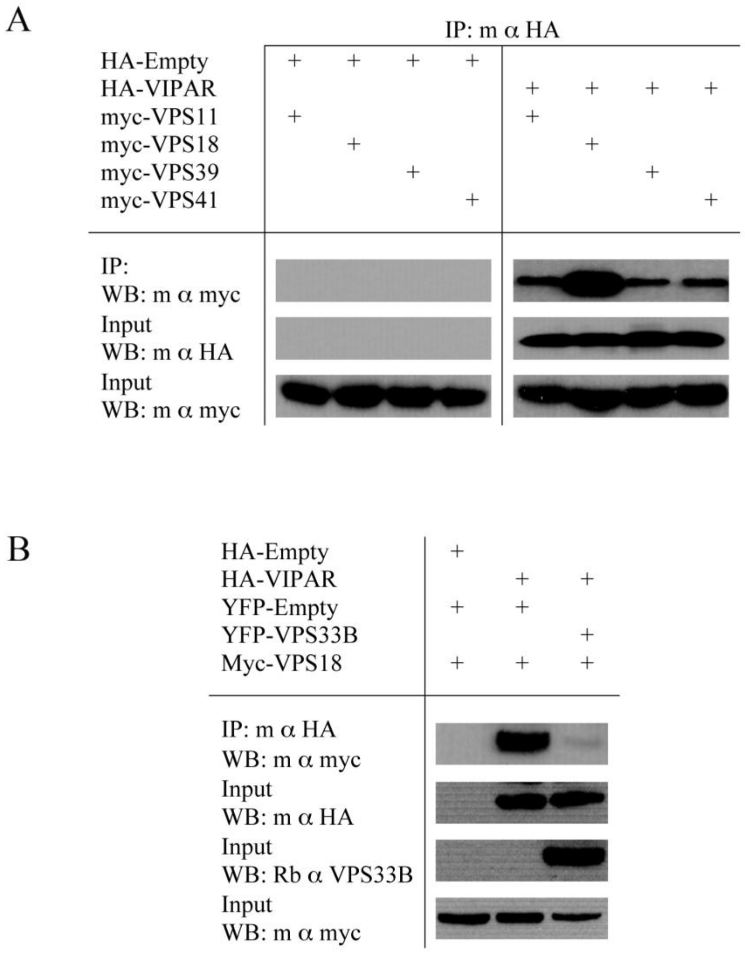

Supp. Figure S3. Co-immunoprecipitation of VIPAR with HOPS complex proteins. (A) HEK293 cells were co-transfected with HA-empty or HA-VIPAR and myc tagged VPS11, VPS18, VPS39 or VPS41. Co-immunoprecipitation experiments revealed interaction between over-expressed VIPAR and HOPS complex proteins. More VPS18 than VPS11, VPS39 and VPS41 was pulled down. Although these proteins were present in the lysates, none were pulled down together with HA-empty. (B) HEK293 cells were co-transfected with HA-empty or HA-VIPAR, YFP-empty or YFP-VPS33B and myc-VPS18. A co-immunoprecipitation experiment showed that VIPAR and VPS18 interacted when the over-expressed VPS33B was absent. In presence of over-expressed VPS33B substantially less VPS18 was pulled down.

**Gene and Mutation**	**Epitope-tagged Construct**
Wild-type *VPS33B*	YFP-VPS33B
VPS33B c.89T>C	YFP-VPS33B(L30P)
VPS33B c.1225+5G>C	YFP-VPS33B(c.1225+5G>C)
Wild-type *VIPAR*	Myc-VIPAR
VIPAR c.638T>C	Myc-VIPAR(L213P)

Supp. Table S1. Epitope-tagged constructs modeling patient mutations

Full length wild-type *VPS33B* in *pEYFP-C3*and *VIPAR* in *pCMV-myc* were used for overexpression of the encoded proteins in co-localization studies. Patient mutations were modelled by site-directed mutagenesis of the wild-type constructs.
